# Correction: Aleem et al. Whole-Genome Identification of APX and CAT Gene Families in Cultivated and Wild Soybeans and Their Regulatory Function in Plant Development and Stress Response. *Antioxidants* 2022, *11*, 1626

**DOI:** 10.3390/antiox14020229

**Published:** 2025-02-18

**Authors:** Muqadas Aleem, Saba Aleem, Iram Sharif, Maida Aleem, Rahil Shahzad, Muhammad Imran Khan, Amina Batool, Gulam Sarwar, Jehanzeb Farooq, Azeem Iqbal, Basit Latief Jan, Prashant Kaushik, Xianzhong Feng, Javaid Akhter Bhat, Parvaiz Ahmad

**Affiliations:** 1 Department of Plant Breeding and Genetics, University of Agriculture, Faisalabad 38040, Pakistan; muqadas.aleem@uaf.edu.pk (M.A.); azeem.iqbal@uaf.edu.pk (A.I.); 2 Center for Advanced Studies in Agriculture and Food Security (CAS-AFS), University of Agriculture, Faisalabad 38040, Pakistan; 3 Barani Agricultural Research Station, Fatehjang 43350, Pakistan; sabaaleem22@gmail.com (S.A.); imran.arid1984@gmail.com (M.I.K.); amnabatool22@gmail.com (A.B.); 4 Cotton Research Station, Ayub Agricultural Research Institute, Faisalabad 38000, Pakistan; iramsharif695@yahoo.com (I.S.); sarwar87@yahoo.com (G.S.); jehanzeb1763@hotmail.com (J.F.); 5 Department of Botany, University of Agriculture, Faisalabad 38040, Pakistan; maidaaleem357@gmail.com; 6 Agricultural Biotechnology Research Institute, Ayub Agricultural Research Institute, Faisalabad 38000, Pakistan; rahilshahzad91@gmail.com; 7 Department of Clinical Pharmacy, College of Pharmacy, King Saud University, Riyadh 11451, Saudi Arabia; basitlatief@gmail.com; 8 Independent Researcher, 46022 Valencia, Spain; prashantumri@gmail.com; 9 Zhejiang Lab, Hangzhou 311121, China; 10 Key Laboratory of Soybean Molecular Design Breeding, Northeast Institute of Geography and Agroecology, Chinese Academy of Sciences, Changchun 130012, China; 11 International Genome Center, Jiangsu University, Zhenjiang 212013, China; 12 Botany and Microbiology Department, College of Science, King Saud University, 8, Riyadh 11451, Saudi Arabia; 13 Department of Botany, GDC, Pulwama 192301, India

In the original publication [1], there was an error regarding the affiliation for author Prashant Kaushik. Specifically, affiliation number 8 (Instituto de Conservación y Mejora de la Agrodiversidad Valenciana, Universitat Politècnica de València, 46022 Valencia, Spain) has been removed from the affiliation list, as per the institution’s request. Dr. Prashant Kaushik will be listed as Independent Researcher. The eighth affiliation for Prashant Kaushik should be as follows:

^8^ Independent Researcher, 46022 Valencia, Spain; prashantumri@gmail.com

The authors state that the scientific conclusions are unaffected. This correction was approved by the Academic Editor. The original publication has also been updated.
