# Supplementary material for: Whole-Genome Identification of APX and CAT Gene Families in Cultivated and Wild Soybeans and Their Regulatory Function in Plant Development and Stress Response
Source: Antioxidants (Basel). 2022 Aug 22;11(8):1626. doi: 10.3390/antiox11081626 (PMC9404807; doi:10.3390/antiox11081626)
Supplement: Supplementary file 1 [file antioxidants-11-01626-s001.zip › Supplementary tables Legends.pdf]

Table S1. Systematic approach followed to identify the APX genes in cultivated and wild soybeans.

Table S2. Systematic approach followed to identify the CAT genes in cultivated and wild soybeans.

Table S3. Cis-regulatory elements identified in the APX gene promoters.

Table S4. Cis-regulatory elements identified in the CAT gene promoters.

Table S5. Collinearity analysis of the *APXs* and *CATs* gene pairs.

Table S6. RNA-seq data of fourteen soybean tissues for *APX* and CAT genes freely available at SoyBase.

Table S7. *APX* and *CAT* genes expression dataset (RNA-seq) under WD, HS and WD+HS stresses publicly available at SoyBase.

Table S8. Primers used for RNA-sequence analysis

Table S9. Wild soybean APX and CAT genes closest homologs in Arabidopsis
